# Real time object detection using LiDAR and camera fusion for autonomous driving

**DOI:** 10.1038/s41598-023-35170-z

**Published:** 2023-05-17

**Authors:** Haibin Liu, Chao Wu, Huanjie Wang

**Affiliations:** Faculty of Materials and Manufacturing, Beijing University of Technology, 100 Pingleyuan, Chaoyang District, Beijing, 100124 China

**Keywords:** Civil engineering, Mechanical engineering

## Abstract

Autonomous driving has been widely applied in commercial and industrial applications, along with the upgrade of environmental awareness systems. Tasks such as path planning, trajectory tracking, and obstacle avoidance are strongly dependent on the ability to perform real-time object detection and position regression. Among the most commonly used sensors, camera provides dense semantic information but lacks accurate distance information to the target, while LiDAR provides accurate depth information but with sparse resolution. In this paper, a LiDAR-camera-based fusion algorithm is proposed to improve the above-mentioned trade-off problems by constructing a Siamese network for object detection. Raw point clouds are converted to camera planes to obtain a 2D depth image. By designing a cross feature fusion block to connect the depth and RGB processing branches, the feature-layer fusion strategy is applied to integrate multi-modality data. The proposed fusion algorithm is evaluated on the KITTI dataset. Experimental results demonstrate that our algorithm has superior performance and real-time efficiency. Remarkably, it outperforms other state-of-the-art algorithms at the most important moderate level and achieves excellent performance at the easy and hard levels.

## Introduction

Object detection is one of the most important tasks that needs to be handled robustly and accurately in autonomous driving. Understanding the driving environment is a prerequisite for safe path planning and obstacle avoidance. However, it still faces many great challenges. For example, sensors are easily affected by severe weather conditions such as bright sunlight or heavy rain. The detection system may misinterpret the pedestrians as road-free areas and lead to a crash in these situations. Additionally, the volume of input data for object detection is very large, which makes it difficult to meet the real-time and high uncertainty requirements of autonomous driving. Therefore, it is necessary for autonomous driving to conduct further research and achieve reliable and real-time object detection^1^.

In recent years, RGB cameras and LiDAR have been the most widely used sensors for object detection. The camera works at fast capture rates and provides dense texture information. However, it is difficult to directly detect the shape and position of objects. Meanwhile, as a passive sensor, the camera is easily affected by variations in the amplitude and frequency of light waves. These issues affect how information gathered from the environment is transformed into images^2,3^. A reliable detection unit should be robust enough to handle changes in light intensity. Compared with the RGB camera, LiDAR detects surrounding environments using lasers, which are less affected by the light conditions of surrounding environments. As a result, the distance and shape of objects can be accurately measured. However, compared with the feature-rich information of an RGB image, the point clouds are very sparse, even with the high-resolution LiDAR^4,5^. Therefore, it is important to figure out how to fuse feature-rich information from RGB images with sparse but reliable depth information from LiDAR point clouds. In this way, the fused data contains rich semantic information, accurate distance information, and is less susceptible to light variations, which can improve the reliability of the object detection unit.

In this paper, we focus on how to fuse data from two types of sensors to achieve better detection performance. The main contributions of this paper are threefold: (1) The Siamese network is constructed as the feature encoder based on Yolo-v5 for object detection. (2) The multi-modality data is fused by applying the feature-layer fusion strategy and designing the Addlayer block. (3) The 3D point clouds are converted into 2D depth images to improve real-time efficiency by reducing data volume.

The remainder of this paper is organized as follows: “Related works” briefly reviews the related works for object detection with different types of sensors. “Methods” details the fusion strategy, the Siamese network architecture, and the point cloud preprocessing. “Results and discussion” demonstrates the experimental results and analyzes the effectiveness of the proposed algorithm. And “Conclusion” concludes this paper and introduces the future work.

## Related works

Autonomous vehicles can detect and recognize their surroundings by using a variety of sensors, including camera, LiDAR, or multi-sensor fusion.

In the field of camera-based object detection, Sinan et al.^6^ investigated the image quality and the object detection accuracy rate under extremely harsh weather conditions. Adaptive weighting was used to make decisions that improve data reliability. Ponn et al.^7^proposed a modeling approach based on investigated influence factors and developed a Shapley Additive Explanations (SHAP) approach to analyze and explain the performance of various object algorithms. Fu et al.^8^ developed an object detection CNN to detect camera-based basketball scoring (BSD) and frame different motions. The "you only look once" (YOLO) model was implemented to locate the basketball hoop location, and motion detection was utilized to detect object motion in the basketball hoop. Lee et al.^9^ proposed a novel YOLO architecture with adaptive frame control (AFC) to deal with resource constraints on embedded systems efficiently.

In the field of LiDAR-based object detection, Meyer et al.^10^ developed LaserNet, a fully convolutional neural network that generates multimodal distribution 3D prediction boxes for each point in the point cloud. These distributions were fused to generate predictions for each object. Shi et al.^11^ proposed PointRCNN to generate 3D proposal boxes on spatial points. The coordinates of points are normalized to better learn local spatial features. Ye et al.^12^ constructed a novel one-stage network, the Hybird Voxel Network (HVNet). This network was used by voxel feature encoders (VFE) to solve the problem of fusing different-sized voxels at the point-wise level. Ye et al.^13^ introduce a novel network called the Shape Attention Regional Proposal Network (SARPNET). It deployed a new feature encoder to remedy the sparsity and inhomogeneity of point clouds and embodied an attention mechanism to learn the 3D shape of objects. Fan et al.^14^ proposed an anchor-free LiDAR-based object detector called RangeDet and designed three components to address the issues of previous works. Experimental results obtained using the large-scale Waymo Open Dataset (WOD) demonstrated that it outperformed other methods by a large margin.

In the field of multi-sensor-based object detection, Li et al.^15 ^developed a 3D detection model named DeepFusion to fuse camera features with deep lidar features to perform object detection. This model was proposed based on two novel techniques: Inverse Aug and Learnable Align. The results showed that it achieved better performance. Liu et al.^16 ^proposed a deep neural network named FuDNN to fuse LiDAR–camera. A 2D backbone was used to extract image features, and PointNet++ was used to extract pointcloud features. Then a sub-network was designed to fuse these two modality model features to detect objects. Zhong et al.^17^ briefly reviewed the methods of fusion and enhancement for LiDAR and camera sensors in the fields of depth completion, semantic segmentation, object detection, and object tracking. Xu et al.^18^ proposed a novel two-stage approach named FusionRCNN. It fused sparse geometry information from LiDAR with dense texture information from the camera in the Regions of Interest (RoI). The experiments showed that this kind of popular detector could provide significant boosts.

Overall, camera-based object detection algorithms are easily affected by changes in light intensity. As a result, they are unstable when working in real environments. Besides, their detection accuracy rate for occluded targets is not high enough. The accuracy rates of LiDAR-based object detection algorithms are relatively low because LiDAR cannot provide sufficient feature information due to the sparsity of the point clouds. In contrast, LiDAR-camera-based fusion algorithms usually render point clouds with RGB images. They can hardly meet the real-time requirements because processing large volumes of 3D data takes a long time. In this paper, the accurate position information of LiDAR and the dense texture information of the camera are fused at the feature layer. A Siamese network architecture is proposed to process multi-modality data and perform object detection. Compared with the state-of-the-art algorithms, our algorithm achieves the best comprehensive performance in accuracy and efficiency.

## Methods

This section outlines the proposed object detection algorithm. Firstly, the point clouds are converted into depth images, which reduces the data volume and improves the real-time performance. Then, a Siamese network architecture is constructed with two parallel same branches to process two types of images in a convolutional way to get feature maps. Finally, we introduce feature-layer fusion strategy and design a cross feature fusion block to fuse the feature maps from multi-modality data^10^.

### Point cloud preprocessing

**Figure 1:**
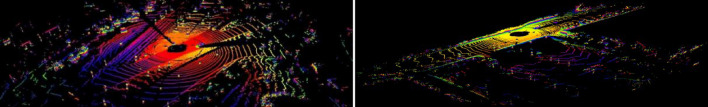
3D point clouds captured by LiDAR.The left figure is one of a 3D point cloud captured by 64-channel LiDAR in the real world. The right figure is another 3D point cloud captured in different scenes. They are visualized by the software Mavayi 4.5.0 (https://anaconda.org/menpo/mayavi) from the KITTI dataset.

As shown in Fig. 1^19^, there are two examples of raw point clouds from the KITTI dataset. It is obvious that raw point clouds are usually disordered and unstructured, which makes them difficult to deal with directly. Traditional preprocessing steps, such as voxel meshing, masking, or vectorization, are usually applied for normalizing. Normalized point clouds, on the other hand, are still 3D modality data that will take a long time to process and cannot meet the real-time requirements of autonomous vehicles.

In this paper, point clouds are converted into depth images to reduce the data volume, which can improve the real-time performance of the detection module. The conversion procedures are as follows: (1) Projecting raw data from the LiDAR coordinate system to the camera coordinate system through spatial rotation and translation by Eq. (1). (2) Transferring the projected data from the camera coordinate system to the image coordinate system through transmission projection by Eq. (2). (3) Transforming the transferred data from the image coordinate system to the pixel coordinate system through scaling and translation by Eq. (3)^20^.

1 $$\left[ {\begin{array}{*{20}c} {X_{{\text{c}}} } \\ {Y_{c} } \\ {Z_{c} } \\ 1 \\ \end{array} } \right] = \left[ {\begin{array}{*{20}c} R & T \\ {0^{T} } & 1 \\ \end{array} } \right]\left[ {\begin{array}{*{20}c} {X_{{\text{L}}} } \\ {Y_{L} } \\ {Z_{L} } \\ 1 \\ \end{array} } \right]$$

where [X_C_, Y_C_, Z_C_] are coordinates of the LiDAR coordinate system, [X_L_, Y_L_, Z_L_] are coordinates of the camera coordinate system, R is the rotation matrix, and T is the translation matrix.

2 $$Z_{{\text{C}}} \left[ {\begin{array}{*{20}c} x \\ y \\ 1 \\ \end{array} } \right] = \left[ {\begin{array}{*{20}c} f & 0 & 0 & 0 \\ 0 & f & 0 & 0 \\ 0 & 0 & 1 & 0 \\ \end{array} } \right]\left[ {\begin{array}{*{20}c} {X_{{\text{C}}} } \\ {Y_{C} } \\ {Z_{C} } \\ 1 \\ \end{array} } \right]$$

where f is the focal length of camera, [x, y] are coordinates of the image coordinate system.

3 $$\left[ {\begin{array}{*{20}c} {\text{u}} \\ v \\ 1 \\ \end{array} } \right] = \left[ {\begin{array}{*{20}c} \frac{1}{dx} & 0 & {u_{0} } \\ 0 & \frac{1}{dy} & {v_{0} } \\ 0 & 0 & 1 \\ \end{array} } \right]\left[ {\begin{array}{*{20}c} x \\ y \\ 1 \\ \end{array} } \right]$$

where [u, v] are coordinates of the pixel coordinate system, and [u_0_, v_0_] is the origin of the pixel coordinate system.

**Figure 2:**
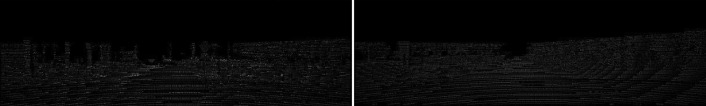
Depth images converted from point clouds.The depth images are generated from 3D point clouds using Eqs. (1)–(3), and they are correlated one by one with the 3D point clouds. They are translated by the software PIL 1.1.7 (https://anaconda.org/free/pil).

**Figure 3:**
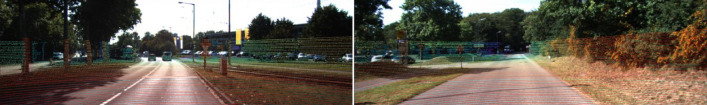
RGB images covered by depth images.The depth images shown in Fig. 2 are overlayed onto the RGB images and aligned with them. They are visualized by the software opencv-python 4.4.0 (https://anaconda.org/sts_dileeppj/opencv-python).

The converted depth images are shown in Fig. 2. It carries very little information because most pixels in the depth image are black. The remaining pixels are composed of detected objects such as vehicles and buildings, while the gray level represents their distances to LiDAR. The depth image is overlaid onto the RGB image to better demonstrate the effectiveness of our conversion. The efficacy of the conversion processes is demonstrated by the fact that the depth images essentially overlap with RGB images, the two overlayed examples on the KITTI dataset are shown in Fig. 3^19^.

### Feature fusion strategy

Multimodal data fusion strategies include three categories: data-layer fusion, feature-layer fusion, and decision-layer fusion. For the data-layer fusion approach, the RGB image and the depth image are converted to tensors and then concatenated in the depth dimension for fusion. The fused tensor contains a large data volume, which brings a huge computation burden to the graphics processing unit (GPU). Under the shallow fusion method, it just simply concatenated raw data without any convolutional processing, which would affect the fusion effect. The decision-layer fusion approach is the highest-level fusion approach for information fusion. The RGB image and depth image are inputted into two independent convolutional neural networks (CNNs) for detection. Then, final decisions are generated by integrating the two results. The detection results generated by the two networks may be mutually exclusive, leading to poor classification performance.

In contrast, the feature-layer fusion approach fuses abstraction feature tensors extracted from two different branches. The data volume of feature tensors is much smaller than that of initial tensors, which can reduce the processing time. It builds connections at multiple convolutional depths between two branches to strengthen the correlation of modality data and improve the data fusion level^21^. The feature-layer fusion method is employed in this paper, and a cross feature fusion block is constructed to achieve it.

### Siamese network architecture

In this paper, RGB image and depth image are used as the inputs of our network, and the feature fusion strategy is employed to fuse the multi-modality data. Therefore, the main challenge is how to construct a neural network structure that can process the two types of data at one time and conveniently extract feature maps from multiple convolutional depths to fuse them. The RGB image and the depth image are two different types of data, but the depth image is projected from the LiDAR point cloud according to the corresponding RGB image captured at the same scene. The depth image and RGB image contain the common feature information of the objects (such as cars, people, etc.) in the environment, and the two types of images have the same size. Therefore, the RGB image and the corresponding depth image are similar. The Siamese neural network performs excellently in processing two similar inputs and has been successfully applied in the fields of face recognition, fingerprint identification, and object tracking. It is composed of a couple of neural networks that can process two different inputs at one time. It can capture more features by maximizing different feature representations through comparing the similarity of the two inputs.

**Figure 4:**
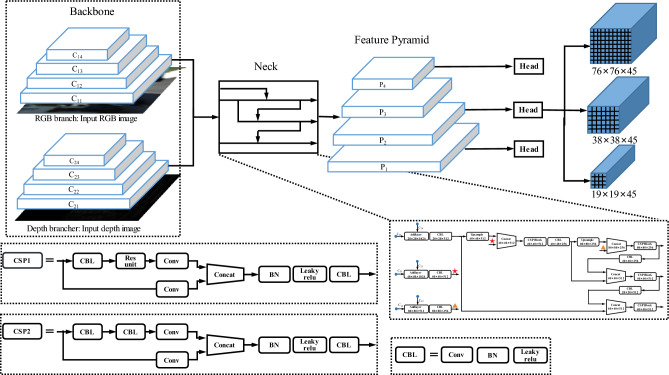
The architecture of the proposed Siamese network. The network structure is composed of three parts: the backbone, the neck, and the head. The backbone consists of two branches: the RGB branch and the depth branch.

For the above reasons, we build a Siamese neural network structure, which includes three key components (as shown in Fig. 4): the Siamese framework, the cross feature fusion block, and two encoder branches.

**Figure 5:**
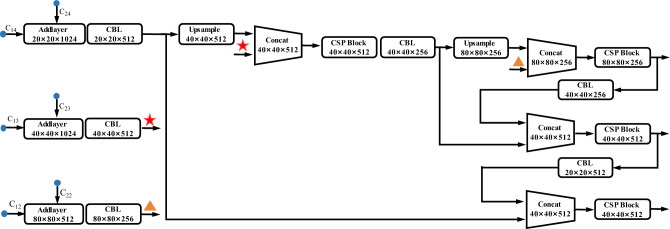
The architecture of the cross feature fusion block. Every logical box represents a network structure; the functions and the output dimensions are listed in the logical box. Stars and triangles are used to notice the connection of two blocks to avoid line interlacing.

1. Constructing Siamese network framework. The RGB image and its corresponding depth image contain texture information and depth information. To ensure that the CNN learns more abstract feature information from multi-modality data, the Siamese network framework is constructed by two parallel same branches to process images at the same time. It extracts more universal features of the objects from multi-modality data, which improves the possibility and accuracy of the object being detected. The two branches jointly train and then test after convergence.
2. Designing cross feature fusion block. The feature fusion block is composed of four Cross Stage Partial (CSP) blocks, three addlayers, four concatenation layers, two upsample layers, and six CBLs (as shown in Fig. 5). Three addlayers are set after the 2nd, 3rd, and 4th CSP blocks of the Siamese network. They extract the feature maps processed by the two branches of the Siamese network and perform an additive operation to fuse the multi-modality data. Addition operations do not change the dimension of feature maps, which means they do not bring additional computational burden. Following the fuse operation, three fusion feature maps ${\mathrm{C}}_{\mathrm{1,22}}$, ${\mathrm{C}}_{\mathrm{1,2}3}$, and ${\mathrm{C}}_{\mathrm{1,2}4}$ are generated, then the feature map ${\mathrm{C}}_{\mathrm{1,2}4}$ is up-sampled to the same size as feature map ${\mathrm{C}}_{\mathrm{1,2}3}$, and these two feature maps are concatenated to generate a new feature map. After passing through a CSP block and a CBL, this new feature map is up-sampled to have the same size with feature map ${\mathrm{C}}_{\mathrm{1,2}2}$ and then they are concatenated. As a result, it can deepen the integration level through multi-time and multi-size fusions. The fusion block fuses the different pieces of information together to increase the texture and context information volume and passes the fusion feature maps back to continue propagating. It adjusts the weights of the two branches according to data from the other branch, which strengthens the correlation of multi-modality data.
3. Employing the CSPDarknet as the backbone of the two branches. The structure of the CSPDarknet is listed in Table 1. CSPDarknet has been used successfully in object detection and has performed admirably in previous work. It mainly consists of five convolutional layers and four CSP blocks. The CSP block draws on the idea of Resnet to build short-cut connections to avoid the vanishing gradient problem in deeper networks. This block also reuses feature maps to reduce the weight parameters.

**Table 1:** The backbone structure of the Siamese network.

	RGB brancher: Input size = 640 × 640 × 3					Depth brancher: Input size = 640 × 640 × 1			
	Type	Filters	Size	Output		Type	Filters	Size	Output
	Convolutional	64	6 × 6/2	320 × 320		Convolutional	64	6 × 6/2	320 × 320
	Convolutional	128	3 × 3/2	160 × 160		Convolutional	128	3 × 3/2	160 × 160
3 ×	Convolutional	64	1 × 1		3 ×	Convolutional	64	1 × 1	
	Convolutional	64	1 × 1			Convolutional	64	1 × 1	
	Bottleneck					Bottleneck			
	Convolutional	128	1 × 1	160 × 160		Convolutional	128	1 × 1	160 × 160
	Convolutional	256	3 × 3/2	80 × 80		Convolutional	256	3 × 3/2	80 × 80
6 ×	Convolutional	128	1 × 1		6 ×	Convolutional	128	1 × 1	
	Convolutional	128	1 × 1			Convolutional	128	1 × 1	
	Bottleneck					Bottleneck			
	Convolutional	256	1 × 1	80 × 80		Convolutional	256	1 × 1	80 × 80
	Convolutional	512	3 × 3/2	40 × 40		Conv	512	3 × 3/2	40 × 40
9 ×	Convolutional	256	1 × 1		9 ×	Convolutional	256	1 × 1	
	Convolutional	256	1 × 1			Convolutional	256	1 × 1	
	Bottleneck					Bottleneck			
	Convolutional	512	1 × 1	40 × 40		Convolutional	512	1 × 1	40 × 40
	Convolutional	1024	3 × 3/2	20 × 20		Convolutional	1024	3 × 3/2	20 × 20
3 ×	Convolutional	512	1 × 1		3 ×	Convolutional	512	1 × 1	
	Convolutional	512	1 × 1			Convolutional	512	1 × 1	
	Bottleneck					Bottleneck			
	Convolutional	1024	1 × 1	20 × 20		Convolutional	1024	1 × 1	20 × 20

The feature pyramid network (FPN) is used as the detection neck to build connections between multi-scale feature maps. Three feature maps ${C}_{\mathrm{1,22}}$, ${C}_{\mathrm{1,2}3}$, and ${C}_{\mathrm{1,2}4}$ are inputted into the FPN architecture for concatenating. In this way, low-level and high-level semantic information are combined to obtain feature maps with rich information.

One-stage detection architecture is introduced to build the detection head, which predicts object classes and locations at the same time. The output has three predictions. Each final prediction has 3(K + 5) output channels, where 3 represents the anchor number, K is the class of the dataset. The following 5 is obtained by adding 4 plus 1, where 4 is the channel of the bounding box localization and 1 is the channel of the objectness prediction score^22^.

### Experimental setup

As one of the largest computer vision evaluation datasets for autonomous driving, KITTI contains HD camera images and related point clouds from Velodyne LiDAR that are collected in urban, rural, and highway settings. It is composed of 7481 training samples and 7518 testing samples^19^. The training and evaluation procedures in this paper are implemented based on this benchmark dataset. The experimental platform is a Lenovo Thinkstation configured with an Nvidia GTX2070Ti GPU, 32 GB of running memory, and the Ubuntu 20.04 operating system. The training and testing details are as follows: The original KITTI training data is split into 3712 training samples and 3769 validation samples for training and validation. The official KITTI test server is utilized to test the performance of our algorithm online. The original images are resized to 640 × 640 pixels. KITTI dataset includes eight classes: tram, misc, cyclist, person (sitting), pedestrian, truck, car, and van. We set up two experiments in this paper. In the first experiment, we mix the eight classes into the three dominant ones: car, pedestrian, and cyclist. In the second experiment, we just evaluated the three dominant classes and removed the rest. The training time is reduced by loading the pre-trained weights file. The training epochs are set to 50, and the batch size is set to 32. The learning rate is initialized at 0.01 and the optimizer is set as SGD.

## Results and discussion

### Experiment 1

**Figure 6:**
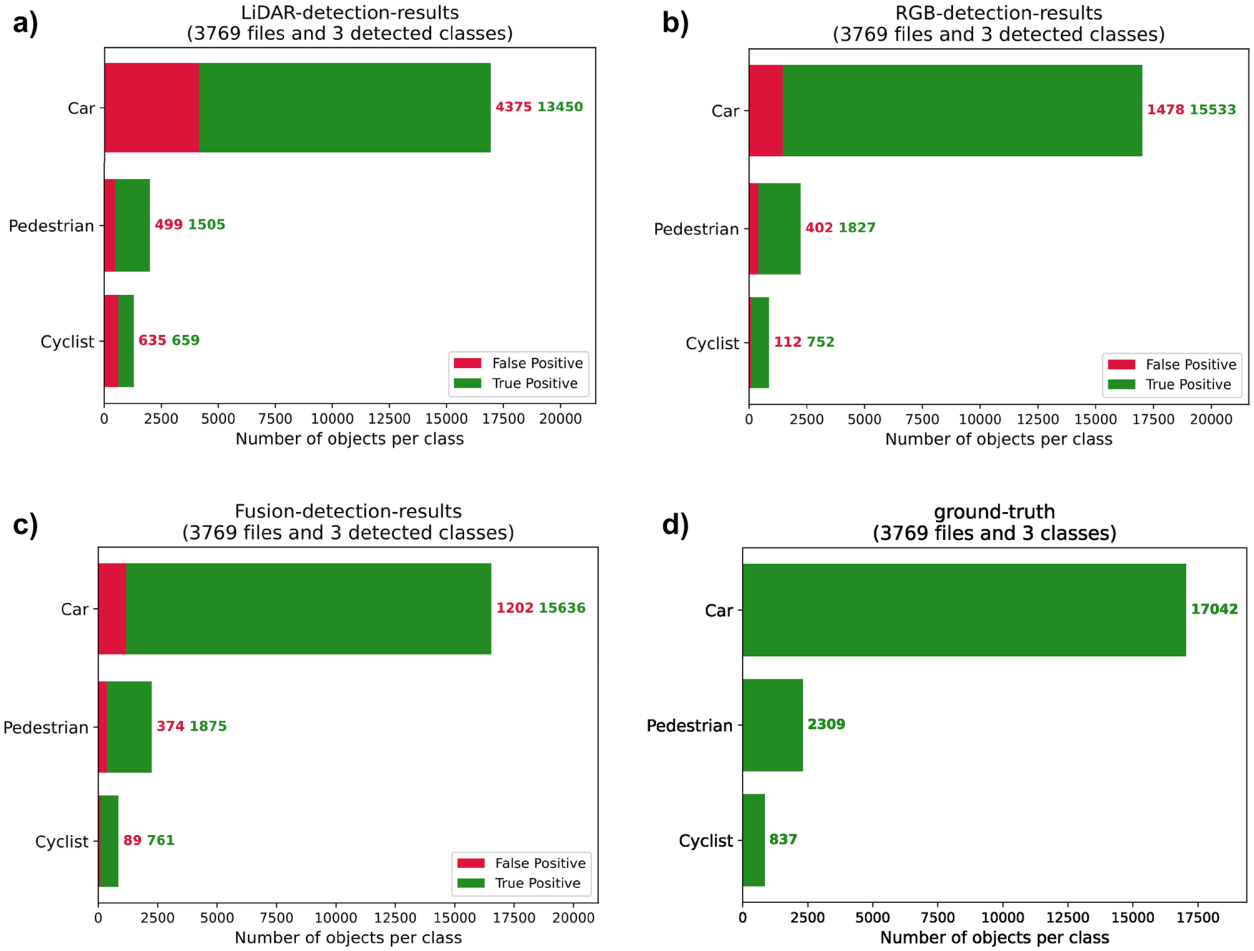
Quantitative analysis results of three algorithms. There are three detected classes: car, pedestrian, and cyclist. Red bars are the false detection samples, and green bars are the true detection samples. (**a**) Lidar-based detection results. (**b**) RGB-based detection results. (**c**) Fusion-based detection results. (**d**) Ground truth.

**Figure 7:**
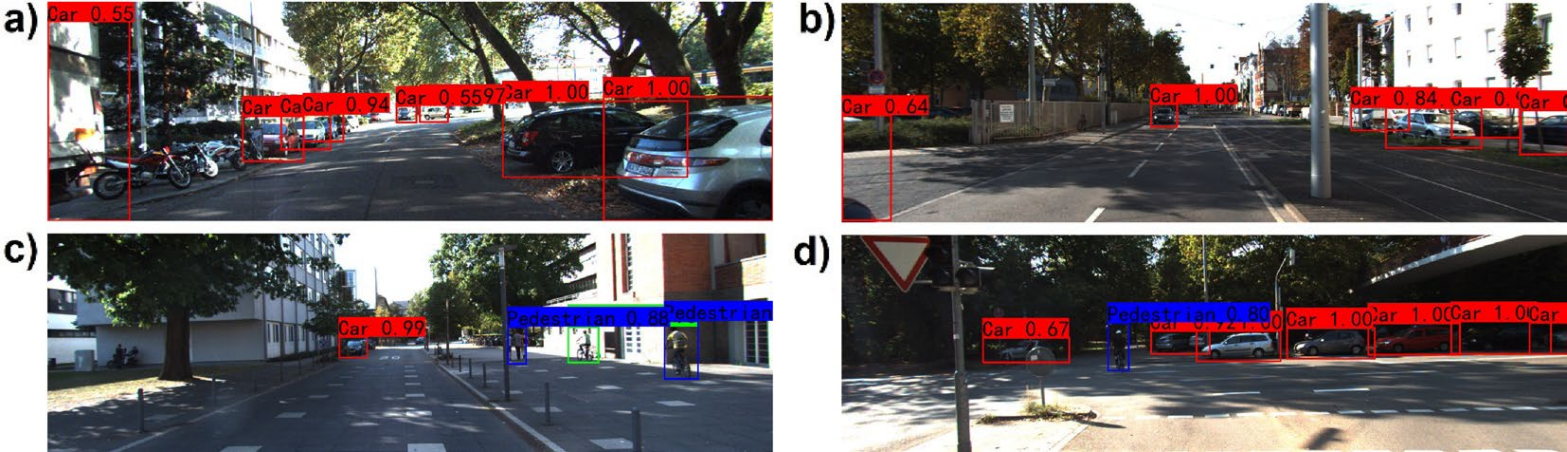
Visual samples of false-positives and false-negatives results of our fusion-based algorithm.

The effectiveness of our proposed fusion algorithm is verified by comparing its training and evaluation performance with single-RGB-based and single-LiDAR-based data. The two single-source-based algorithms share the same backbone as our fusion-based algorithm but have one branch. The comparison results are summarized in Table 2 and Fig. 6. Our fusion-based algorithm exhibits the best overall performance with a mAP of 89.26, followed by the single-RGB-based algorithm with a mAP of 86.70 and the single-LiDAR-based algorithm with a mAP of 74.27. In comparison to the other two single-source-based algorithms, our proposed approach achieves the highest true positive prediction and the lowest false negative prediction. We static the number of the false-negative and false-positive results detected by our fusion algorithm in Table 3. We also visualize some examples of false-negative and false-positive results in Fig. 7. Figure 7a and b illustrate two cases of false-negatives. In Fig. 7a, the left-side residential building was incorrectly identified as a car because the shape and color of the building are very similar to the cargo box of the van. In Fig. 7b, the left-side road surface was incorrectly identified as a car due to the similarity between the shadow of the rear end of a car on the road surface and the actual rear end of a car. In contrast, Fig. 7c and d represent two cases of false-positives. The cyclist on the right side of Fig. 7c and the middle part of the cyclist in Fig. 7d were both incorrectly identified as pedestrians. Although there is a huge difference between the lower halves of the cyclist and pedestrian, the upper halves are highly similar. It is apparent that false-negatives and false-positives occur when the misclassified objects have similar shapes to the correct objects. In future research, we plan to enhance our network architecture by introducing the attention mechanism. This mechanism will facilitate the network in paying more attention to the regions of interest, enabling it to capture a wider range of contextual information. As a result, the network will be better equipped to make accurate predictions based on the historical features of the targets, thus minimizing the occurrence of false-negatives and false-positives.

**Table 2:** Comparison of our fusion-based algorithm with single-source-based algorithms on KITTI val sets.

Sensor	Time(s)	mAP (%)	Car (IOU = 0.7)			Cyclist (IOU = 0.5)			Pedestrian (IOU = 0.5)		
			F1	REC (%)	PRE (%)	F1	REC (%)	PRE (%)	F1	REC (%)	PRE (%)
LiDAR	0.022	74.27	0.81	75.66	78.92	0.73	67.62	78.73	0.68	70.82	65.18
RGB	0.022	86.70	0.91	90.45	91.14	0.88	87.22	89.84	0.77	75.26	79.13
L + R	**0.03**	**89.26**	**0.93**	**91.27**	**94.24**	**0.91**	**89.25**	**92.34**	**0.84**	**86.80**	**81.20**

*PRE* precision, *REC* recall, F1 = 2PR/(P + R), IOU = 0.5.

Significant values are in [bold].

**Table 3:** The number of false-positives and false-negatives detected by our fusion-based algorithm.

	Car (IOU = 0.7)	Cyclist (IOU = 0.5)	Pedestrian (IOU = 0.5)
False-positive	1202	89	374
False-negative	1495	92	285
True-positive	15,636	761	1875

**Figure 8:**
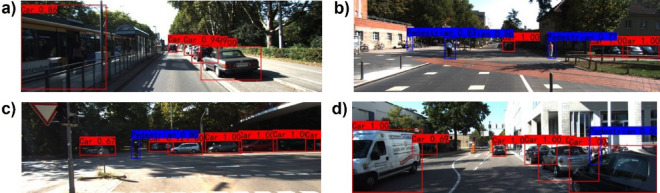
Visual samples of single-RGB-based detection results.

**Figure 9:**
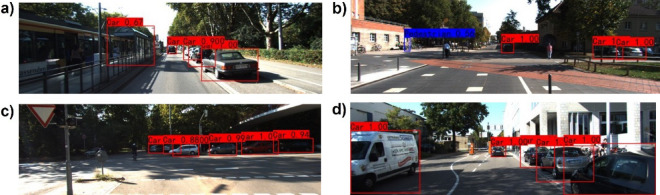
Visual samples of single-LiDAR-based detection results.

**Figure 10:**
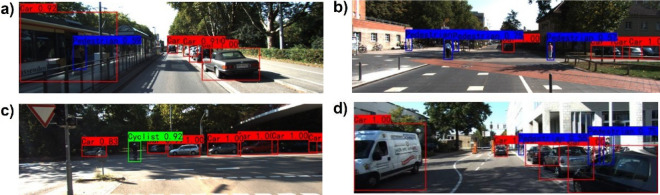
Visual samples of fusion-based detection results.

In order to compare our proposed algorithm with single-source-based algorithms more intuitively, we visualize some detection results on the KITTI dataset^19^ and make a qualitative analysis. Figures 8a and 9a show that the single-RGB-based and single-LiDAR-based algorithms both miss the detection of the pedestrian who is getting off the bus. Figures 8b and 9b illustrate that the single-RGB-based and single-LiDAR-based algorithms falsely detect three cars at the right corner of the image. In Figs. 8c and 9c, the single-RGB-based algorithm misidentifies the cyclist as a pedestrian, while the single-LiDAR-based algorithm misses detecting this object. Figures 8d and 9d show that the single-RGB-based and single-LiDAR-based algorithms both miss the detection of pedestrians occluded by cars. Figure 10 also illustrates that our fusion-based algorithm successfully detects all targets and regresses their locations. In summary, our proposed algorithm performs better than the single-source algorithms, especially in the object detection and position regression of small targets, targets at the border of the image, and occluded targets.

### Experiment 2

**Figure 11:**
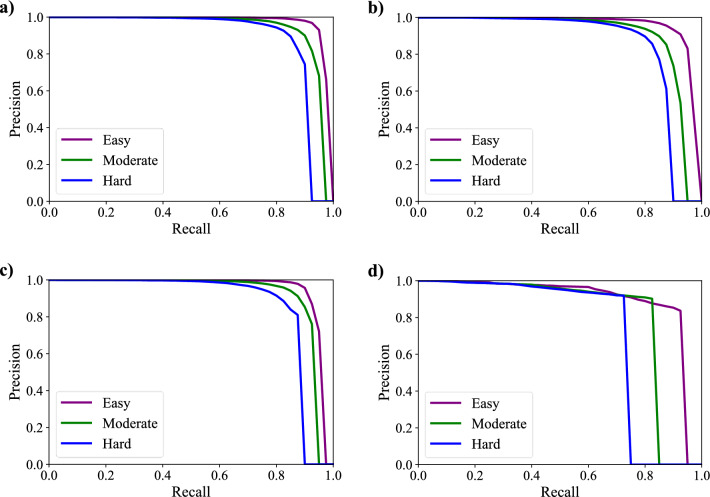
Samples of fusion-based detection results. (**a**) Our detection results. (**b**) AVOD-FPN detection results. (**c**) TAN-Net detection results. (**d**) 3DSSD detection results. The three different metric levels (easy, moderate, and hard) are represented in purple, green, and blue, respectively.

The proposed algorithm is also compared with other state-of-the-art algorithms on KITTI test sets and val sets. To perform evaluations, three different metric levels are defined by KITTI official standards: easy, moderate, and hard. Tables 4 and 5 list the experimental results, while the detection results of four better algorithms are shown in Fig. 11. The running time and environment of each algorithm are presented in Table 6. Table 4 summarizes the performance comparisons between our algorithm and other state-of-the-art algorithms on the official KITTI test sets. It demonstrates that our algorithm is the best in efficiency and achieves the highest score at all easy and moderate levels for car detection compared with LiDAR-based, camera-based, and LiDAR-camera-fusion-based algorithms. Although the detection performance at the hard level is slightly lower than that of TANet^30^, our algorithm outperforms TANet by 1.94 percent at the most important moderate level. The superior performance benefits from the use of the Siamese network for fusing different source data at multiple-layers. In this way, the model can better learn depth and texture information based on multi-modality data. We also validate the performance of our algorithm with the aforementioned algorithms on KITTI val sets for further validation. As shown in Table 5, our algorithm not only achieves the best comprehensive performance but also ensures real-time detection. It only takes 0.03 s per frame, which is much faster than the other algorithms. Although the detection result of our algorithm at an easy level is slightly lower than that of AVOD-FPN^31^, it still shows high advantages over any other indicator. Traditional LiDAR-based algorithms are unable to meet the real-time requirements in real environments because they take raw point clouds as input for CNN without any preprocessing. In this paper, 3D point clouds are converted into 2D depth images to reduce the data volume, which can improve the detection efficiency without losing depth information. Besides, as shown in Table 7, our algorithm also performs excellently on pedestrian and cyclist detection.

**Table 4:** Comparison with state-of-art methods on test set.

Method	Time (s)	Sensor	Detection-car (Iou = 0.7)		
			Easy	Moderate	Hard
F-Pointnet^23^	0.17	L + R	82.19	69.79	60.59
3D-CVF atSPA^24^	0.06	L + R	89.20	80.05	73.11
CLCOs^25^	0.10	L + R	88.94	80.67	77.15
ImvoxelNet^26^	0.20	L + R	88.80	79.09	69.45
Pointpillars^27^	0.26	L	82.58	74.31	68.99
PointRCNN^28^	0.08	L	86.96	75.64	70.70
3DSSD^29^	0.12	L	88.36	79.57	74.55
TANet^30^	0.04	R	93.67	90.67	**85.31**
AVOD-FPN^31^	0.10	R	94.70	88.92	84.13
Ours	**0.03**	L + R	**94.92**	**92.61**	84.83

Significant values are in [bold].

**Table 5:** Comparison with state-of-art methods on val set.

Method	Time (s)	Sensor	Detection-car (Iou = 0.7)		
			Easy	Moderate	Hard
MV3D^32^	0.24	L + R	86.55	78.10	76.67
F-Pointnet^23^	0.17	L + R	83.76	70.9	63.65
3D-CVF atSPA^24^	0.06	L + R	89.67	79.88	78.47
STD^33^	0.08	L + R	89.71	79.45	78.67
Pointpillars^27^	0.26	L	86.62	76.06	68.91
PointRCNN^28^	0.08	L	89.35	83.69	78.70
3DSSD^29^	0.12	L	89.71	79.45	78.67
TANet^30^	0.04	R	94.57	91.67	82.71
AVOD-FPN^31^	0.10	R	**96.07**	89.72	83.23
Ours	**0.03**	L + R	95.69	**93.41**	**85.54**

Significant values are in [bold].

**Table 6:** Comparison of running time and environment with state-of-art methods.

Method	Time (s)	Environment
F-Pointnet^23^	0.17	GPU @ 3.0 GHz(python)
3D-CVF atSPA^24^	0.06	1 core @ 2.5 GHz(C\C++)
CLCOs^25^	0.10	1 core @ 2.5 GHz(C\C++)
ImvoxelNet^26^	0.20	GPU @ 2.0 GHz(python)
Pointpillars^27^	0.26	1080 Ti GPU
PointRCNN^28^	0.08	GPU @3.5 GHz(Python + C\C++)
3DSSD^29^	0.12	GPU @2.5 GHz(Python + C\C++)
TANet^30^	0.04	GPU @2.5 GHz(Python + C\C++)
AVOD-FPN^31^	0.10	**Tian X (Pascal)**
MV3D^32^	0.24	GPU @2.5 GHz(Python + C\C++)
STD^33^	0.08	GPU @2.5 GHz(Python + C\C++)
Ours	**0.03**	GPU @2.5 GHz(Python)

Significant values are in [bold].

**Table 7:** Performance of our algorithm for pedestrian and cyclist on the KITTI val set.

Method	Time (s)	Sensor	Detection-cyclist (Iou = 0.5)			Detection-pedestrian (Iou = 0.5)		
			Easy	Moderate	Hard	Easy	Moderate	Hard
Ours	0.03	L + R	72.52	54.50	48.82	79.23	65.34	60.57

## Conclusion

This paper proposes a LiDAR-Camera fusion algorithm for real-time object detection. The raw point clouds are converted into depth images through joint calibration of the LiDAR and camera coordinate systems. The Siamese network is constructed based on Yolo-v5 to integrate RGB images and depth images at multiple convolutional layers between two branches. The proposed algorithm is evaluated on the KITTI dataset. Experimental results demonstrate that the algorithm performs excellently in detecting small and occluded objects. It also shows the best overall detection performance compared with other state-of-the-art algorithms. In future work, the possible extensions of the feature fusion architecture will be explored by supervising the data flow between the two branches of the Siamese network. To improve the network's focus on areas of interest and reduce the incidence of false-negatives and false-positives, the attention mechanism will be introduced. Additionally, we plan to embed our algorithm on unmanned ground platforms under real-world conditions and design experiments to further validate its robustness by changing light intensity and other environmental conditions.

## Data Availability

The dataset generated and/or analyzed during the current study are available in the KITTI repository, and can be accessed at [https://www.cvlibs.net/datasets/kitti/raw_data.php]. This dataset is a open source dataset and licensed under the Creative Commons Attribution-NonCommercial-ShareAlike 3.0 Unported License. To view a copy of this license, visit http://creativecommons.org/licenses/by-nc-sa/3.0/ or send a letter to Creative Commons, PO Box 1866, Mountain View, CA 94042, USA.
